# The Budget and Private Enclosed Gardens

**Published:** 1909-10-30

**Authors:** J. Lawrence-Hamilton

**Affiliations:** 30 Sussex Square, Brighton


### Editor's Letter-Box.

### THE BUDGET AND PRIVATE ENCLOSED GARDENS.

Sir,—Though apparently the Government has withdrawn its proposals to tax enclosed private gardens, it still seems advisable that the attention of the public should be drawn to the dangers it has so narrowly escaped from such ill-considered propaganda.

The Budget proposed to tax doubly private enclosed grounds attached to "squares," "gardens," "crescents," and similar collections of houses. It claimed the right to do so, because these sanitary health-giving local lungs are withheld from the greedy speculations of the speculative jerry builder, and that these quiet, peaceful, picturesque areas are not available to the public.

Unfortunately, till quite recently, town planning was neglected by our short-sighted municipal authorities. Indeed, throughout the United Kingdom the intelligent enterprises of far-seeing private individuals alone prevented local over-crowding and over-building by the costly creation —at their own expense—of numerous private enclosed gardens, managed and supported by local residents paying enhanced rates and taxes, because of the privilege of having sole access to these grounds.

Hence, to again tax these houses a second time by taxing their private enclosed gardens would be unjustifiable. It comes to this, that it has been suggested to put an additional tax on picturesque town-planning, tree-planting, hedges, grass lawns, shrubs and flowers, because these embellishments have been provided by private enterprise.

If the taxation of private garden enclosures were granted, then would it not follow that the taxation of houses bordering upon lakes, rivers, and the sea, would also be retaxed, because of their adjoining aquatic air spaces? Such taxes on air spaces would be even more injurious to public health than the old tax on windows which were guilty of admitting sunlight, warmth, and air.

To avoid noise and vibrations created by "tubes," trams, trains, traffic, and motor-cars, I have long resided in a secluded, sheltered "square" in Brighton, which, in addition to extensive elegant enclosed private gardens and sea terraces sloping down to the beach below, has a unique financial history that has hitherto escaped publicity.

These Sussex Square Gardens, constituting the Kemp Town Enclosures, are a model miniature marine garden city, being Brighton's chiefest adornment.

Early in the nineteenth century Thomas Read Kemp (1781 to 1844) converted his Black Rock Farm and adjacent property into a magnificent estate, named, after him, Kemp Town, the attraction and adornment of which are the enclosed picturesque "Sussex Square" Gardens, covering some ten acres, with their sea-terraces and grass-covered slopes, extending to upwards of half a mile along the beach.

The Park was planned by Sir Joseph Paxton, including beneath the main road or sea front a tunnel connecting the gardens with the marine terraces below. Under the highway on each side of the tunnel is a cleverly concealed cottage—like a cave dwelling—its roof being underneath the public footpath above. The top of the "Square" is nearly 120 feet above the sea's level.

Sheltered from northerly winds by an ivy-clad wall some 35 feet high, the upper and lower marine terraces contain a dozen capacious arches, providing excellent shelter and summer houses. This domain is only accessible to the residents of the 103 houses constituting this enclosed estate, the quiet and privacy of which pleased the King in 1908 when staying at the Duke of Fife's residence at the corner of "Sussex Square."

By 1837 Kemp's magnificent town-planning punished him with a loss exceeding £500,000. He died in comparative poverty in Paris in 1844.

For nearly a century these enclosures have been and are maintained by subscriptions from 103 owners and occupiers of houses on the estate. Their residents have paid extra local rates and taxes for access to the grounds, which yield neither income nor financial profit.

Of course this site can never be developed for building purposes, and to treat and tax it as land withheld from the market with a view to increased rent, is absurd. Indeed, already upwards of £40,000 for wages and materials have been distributed for the upkeep of this park by the owners and occupiers of houses upon this estate, where empty uninhabited houses also pay annual subscriptions for the maintenance of the gardens.

J. Lawrence-Hamilton, M.R.C.S.

30 Sussex Square, Brighton.

